# Supplementary figures and images for: Exploring Cationic Guar Gum: Innovative Hydrogels and Films for Enhanced Wound Healing
Source: Pharmaceutics. 2024 Sep 22;16(9):1233. doi: 10.3390/pharmaceutics16091233 (PMC11435176; doi:10.3390/pharmaceutics16091233)

## Supplementary File

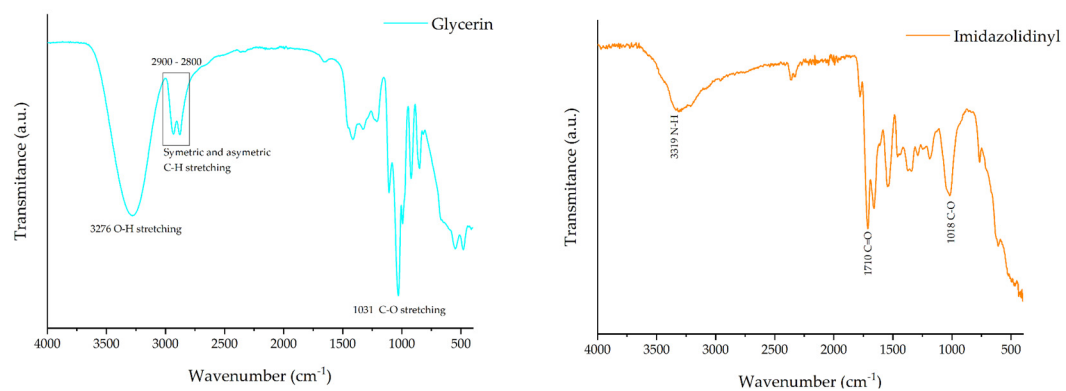

**Figure S1.** FTIR of Glycerin and Imidazolidinyl.

Supplement: Supplementary file 1 [file pharmaceutics-16-01233-s001.zip › pharmaceutics-3188470-supplementary.pdf]
